# Complicated malaria and other severe febrile illness in a pediatric ward in Libreville, Gabon

**DOI:** 10.1186/1471-2334-12-216

**Published:** 2012-09-13

**Authors:** Marielle Karine Bouyou-Akotet, Denise Patricia Mawili-Mboumba, Eric Kendjo, Ariane Eyang Ekouma, Omar Abdou Raouf, Edouard Engohang Allogho, Maryvonne Kombila

**Affiliations:** 1 Department of Parasitology-Mycology, Faculty of medicine, Université des Sciences de la Santé, B.P.4009, Libreville, Gabon; 2 Malaria Clinical and Operational Research Unit, Libreville, Gabon; 3 Pediatric ward A, Centre Hospitalier de Libreville, Libreville, Gabon

## Abstract

**Background:**

Although a substantial decline of *Plasmodium falciparum* infection is observed in Africa following implementation of new control strategies, malaria is still considered as the major cause of febrile illness in hospitalized African children. The present study was designed to assess the management of febrile illness and to determine the proportion of children with febrile illness hospitalized for primary diagnosis of malaria who had confirmed complicated malaria after implementation of new malaria control strategies in Libreville, Gabon.

**Methods:**

Demographic, clinical and biological data from hospitalized children with fever or a history of fever, with a primary diagnosis of clinical malaria, aged less than 18 years old, who benefited from hematological measurements and microscopic malaria diagnosis, were recorded and analyzed during a prospective and observational study conducted in 2008 in the Centre Hospitalier de Libreville.

**Results:**

A total of 418 febrile children were admitted at hospital as malaria cases. Majority of them (79.4%) were aged below five years. After medical examination, 168 were diagnosed and treated as clinical malaria and, among them, only 56.7% (n = 95) had *Plasmodium falciparum* positive blood smears. Age above five years, pallor, Blantyre Coma Score ≤2 and thrombocytopenia were predictive of malaria infection. Respiratory tract infections were the first leading cause of hospitalization (41.1%), followed by malaria (22.7%); co-morbidities were frequent (22%). Less than 5% of suspected bacterial infections were confirmed by culture. Global case fatality rate was 2.1% and 1% for malaria. Almost half (46%) of the children who received antimalarial therapy had negative blood smears. Likewise, antibiotics were frequently prescribed without bacteriological confirmation.

**Conclusions:**

The use of clinical symptoms for the management of children febrile illness is frequent in Gabon. Information, training of health workers and strengthening of diagnosis tools are necessary to improve febrile children care.

## Background

In Africa, one in six children dies before its fifth birthday from treatable conditions such as pneumonia, gastroenteritis and malaria [1]. In 2009, the World Health Organization (WHO) estimated that among the 761,000 deaths attributable to malaria, more than 80% occurred in African children less than five years old [2]. During recent decades, malaria has become a default diagnosis for acute febrile illness in many African settings though is presently declining [3]. Moreover, clinical manifestations of complicated forms of malaria, which include mainly cerebral malaria and severe anemia, usually overlap those of other severe febrile illness in endemic countries.

Presumptive treatment of fever was recommended by WHO for a long time. However, such a strategy is no longer satisfactory because of its poor specificity, leaving non-malarial febrile patients without an appropriate treatment. In fact, evidence suggests a higher mortality in children diagnosed and treated for malaria without microscopic confirmation compared to those who benefited from microscopically confirmed malaria [4]. The biological diagnosis of malaria is still not routinely done by health staff from many endemic countries, even when it is available [5]. In Gabon, where a substantial decline of malaria prevalence in Libreville, the capital city, has been observed, little is known about the suitability of treatment of patients in the pediatric wards in public hospitals |5]. Indeed, a threefold reduction of malaria cases was observed in the country [6,7]. A shift toward a higher susceptibility of children older than five years was also noted for uncomplicated malaria, suggesting an epidemiological transition of the infection in the country. The frequency of complicated malaria should also be influenced by interventions. Therefore, the decline of malaria prevalence can also result in a parallel decrease of, or influence the frequency of cerebral malaria (CM) and severe malarial anaemia (SMA) as reported in north-eastern Tanzania and in rural Kenya [8,9].

The aim of the present study was to assess the management of febrile illness and to determine the proportion of children with febrile illness hospitalized for primary diagnosis of malaria who had confirmed complicated malaria, at the Centre Hospitalier de Libreville, the main public health centre of Gabon, after implementation of new malaria control strategies.

## Methods

### Study site

The survey took place in Libreville, the capital city of Gabon where more than 40% of the population lives. In this urban area, the climate is equatorial; malaria prevalence among febrile children was 15% in 2008, predominantly caused by *P.falciparum*. Annual entomological inoculation rate is 33.9 and *Anopheles gambiae s.s.* is the main vector [10]. The Centre Hospitalier de Libreville (CHL) is the largest public hospital of Gabon; it has two pediatric wards where patients aged between 30 days and 17 years old are admitted. During the study period, due to the closing of the second ward (22 beds) for its rebuilding, the recruitment was performed in the main pediatric ward which has a capacity of 35 beds with about 1440 hospitalized children per year. The clinical care of these patients is performed by medical doctors and pediatricians. Children are visited daily by one of the medical staff member.

The Malaria Clinical and Operational Research Unit (MCORU) is a branch of the Department of Parasitology of the Medicine Faculty, located in the CHL. In this unit, all febrile in and out-patients from the pediatric and emergency wards routinely benefit from free malaria diagnosis based on microscopic examination.

### Study design

This was a prospective and observational pilot study carried out from June to December 2008, in Libreville. This period covers the dry and the rainy seasons, as well as the period at which the maximum number of consultations is observed at the CHL, i.e. after returning from holidays from remote areas, at the time of the start of the new school year.

Data from children aged less than 18 years old, with fever (rectal temperature ≥ 38.0°C) or a history of fever during the 24 hours preceding the consultation, hospitalized for primary diagnosis of clinical malaria, who benefited from hematological measurements and *P. falciparum* microscopic detection, were prospectively collected and analyzed.

### Data collection

Oral consent was obtained from parents or guardians in order to complete the demographic and medical history sections on a case report form (CRF) by questioning the parents if necessary. Recorded demographical data were date of birth, residence, marital and employment status of the parents or tutors. The medical history section comprised the following: previous malaria, drug intake, blood transfusion, vaccination status, use of bednet, asthma, confirmed sickle cell trait, duration of illness. The clinical data collected for the current disease were the symptoms leading to the consultation; the physical examination included the assessment of weight, general physical state, rectal temperature, respiratory frequency, mucosal coloration, nutritional (weight for age z-score) and hydration status. The nutritional status was classified as follows: children with a Z score > -2 were classified as normal nourished, children with a Z score < −2 and > = −3 as moderately malnourished, and children with a Z score < −3 as severely malnourished. Additional investigations, based on clinical symptoms and blood count if judged necessary by physicians, were chest radiography, cerebro-spinal fluid (CSF), stool and sputum analysis, blood, CSF and urine culture, HIV-antibody detection, tuberculin intra-dermal reaction.

Based on various criteria, the following diagnosis were defined: malaria (fever with positive blood slide), suspected lower respiratory tract infection (fever associated with respiratory symptoms and abnormal X-rays), meningitis (fever with neurological symptoms and abnormal CSF if analysis performed), urinary infection (fever in presence of suprapubic pain and pathogen identified in urine culture when performed), gastroenteritis (fever with vomiting and diarrhea), pulmonary tuberculosis (suspected low respiratory tract infection and positive expectoration associated with a positive intradermal reaction).

Laboratory investigations allowed the definition and classification of hyperparasitemia if parasite count >250,000/μL; of anaemia classified as severe if Hb < 5.0 g/dL, moderate if 5.0 < Hb < 8.0 g/dL and low if 8.0 ≥ Hb < 10.9 g/dL; of leucocytosis defined by taking into account the rate of leucocytes according to the age; of thrombocytopenia when platelet count was below 150.10^3^/μL.

### Treatments

The therapeutic management was recorded as well as the clinical evolution on the CRF. All children enrolled were hospitalized and malaria was treated with intravenous quinine (25 mg/kg/day,) followed by oral quinine when patient was able to swallow. Febrile children received intravenous paracetamol or suppositories (60 mg/kg/day). Seizures were controlled with diazepam, severe anaemia and not tolerated moderate anaemia were corrected by transfusion of packed red cells. Other treatments were intravenous fluid for severe dehydration, antibiotics for suspected bacterial infections, nasal oxygen at 6 L/minute for respiratory distress.

### Consideration

The study was approved by the public health ministry of Gabon. Study aims were clearly explained to the paediatric ward staff. Children's parents or guardians were informed about the protocol, and their oral consent was required prior to data collection. Although it was a non invasive study, this oral consent was sought from the parents/guardians in order to obtain medical history and to use the demographic, clinical and biological data and other results of other investigations that were reported on the CRF. Data were kept as confidential.

### Statistical analysis

All data were entered and cleaned using Epi-info version 3.3.2 (2005 CDC Atlanta).

Variables were summarized as frequencies and percentages, means and standard deviation, or medians and interquartile ranges, as appropriate. They were compared with the use of the chi-square test, Anova, or non-parametric tests as appropriate. Median with interquartile ranges was used for age and parasitaemia. We performed logistic regression to assess the effect of various confounding factors and potential determinants *P.falciparum* malaria. Analysis resulting in values of *p* < 0.05 were considered significant. All reported *p*-values are two-tailed.

## Results

### Study population

During the year 2008, 4684 children were screened at the MCORU, 2521 were hospitalized in the different wards. From June to December, 804 patients were admitted in the pediatric ward, 431 febrile of them were considered as malaria cases and benefitted from both malaria diagnosis and haematological count. Thirteen (3.0%) patients were excluded from the analysis due to missing data on the CRF. Among the remaining patients, 235 were referred by the outpatient unit and 183 came from the emergency ward. The median age of patients was 24 [12–48] months; the youngest was one month and the oldest 192 months. Age was distributed as follows: <6 months (n = 44, 10.5%), 6 to 18 months (n = 147, 35.2%), 19 to 36 months (n = 110, 26.3%), 37–59 months (n = 31, 7.4%), 60–120 months (n = 66, 15.8%) and above 120 months (n = 20; 4.8%). More than half of the children (n = 257; 61.5%) were aged 6 to 36 months. The sex ratio was 1.2.

### Medical history and clinical data

All patients were referred with a primary diagnosis of malaria; 38 (9.1%) were sickle cell trait carriers and 32 (7.6%) had a history of asthma. One third (n = 138; 33%) of patients were not well vaccinated according to the Enlarged Program of Immunization and 66.5% (n = 278) used bednets. Main reasons, for consultation aside from fever or history of fever, were asthenia, abdominal symptoms, cough and seizures (Table 1). Five (1.2%) children were admitted with a BCS below 2. Less than 10% (n = 27) and 32% (n = 134) of children were administrated antibiotic or antimalarial drugs before consultation, respectively.

**Table 1:** Hospital recorded diagnosis based on patient clinical features

**Characteristics**	**Median**	**Interquartile range**
**All admissions (n = 418)**		
Age in months,	24.0	[12–48]
Duration of illness in days	3.0	[2–5]
Temperature in °C	38.9	[38.0-39.3]
	**N**	**(%)**
Female	192	(46)
Pallor	211	(51)
**Main reasons for consultation**		
Asthenia	193	(46)
Repeated vomiting	151	(36)
Diarrhoea	120	(29)
Abdominal pain	32	(8)
Cough	118	(28)
Dyspnea	32	(8)
Seizures	73	(17)
Blantyre Coma Score < 5	62	(15)
**Diagnosis of lower respiratory tract infection**^**a**^**(n = 172/418)**		
Cough	91	(53)
Rattling	79	(46)
Deep breathing	14	(8)
Pneumonia syndrome	40	(23)
Abnormal x-ray	151	(88)
**Diagnosis of gastro-enteritis**^**a**^**(n = 74/418)**		
Vomiting	63	(85)
Dehydratation	65	(88)
Diarrhoea	67	(90)
**Diagnosis of meningitis**^**a**^**(n = 16/418)**		
Vomiting	6	(38)
BCS ≤ 4	8	(50)
Seizures	7	(44)
Neck stiffness	7	(44)
Abnormal CSF^b^	5/7	(71)

^a^: cases can appears in many categories; ^b^: cerebro-spinald fluid.

Median temperature was 38.9°C, and pallor was observed in half of the patients (51%; n = 211) (Table 1). Malnutrition, either moderate (n = 26) or severe (n = 15), concerned 41 (9.8%) patients. Frequency of severe malnutrition was of 8.8% (n = 13/147) among children aged between six and 18 months.

Thick smears and hematological analysis were systematically performed for hospitalized patients, X-rays were frequently prescribed (n = 299, 71.5%), whereas CSF and urine analysis, as well as stools and blood cultures, were realized for 4.5% (n = 19), 2.6% (n = 11), 1.7% (n = 7) and 3.1% (n = 13) of patients respectively. Tuberculin intra-dermal reaction and HIV testing were requested for 54 (12.9%) and 29 (6.9%) patients respectively.

**Figure 1:**
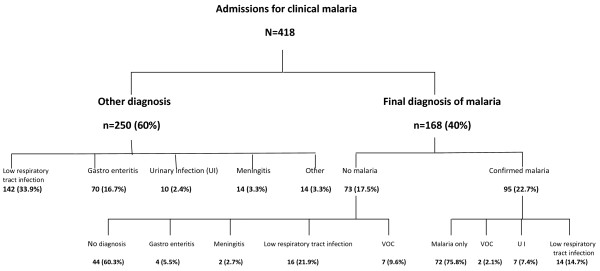
Distribution of main fever-attributable etiologies among children admitted for primary diagnosis of malaria.

After all investigations, lower respiratory tract infection (n = 172, 41.1%), malaria (n = 168, 40.2%), gastro enteritis (n = 74, 17.7%) and meningitis (n = 16, 3.8%) were the main recorded final diagnosis (Table 1 and Figure 1). Pulmonary tuberculosis was diagnosed in 16 (9.3%) patients. HIV infection was detected in nine patients. Bacterial gastro-enteritis was confirmed in five patients, due to *Escherichia coli* (n = 4) and *Shigella* (n = 1). Among the 168 patients with a final diagnosis of clinical malaria, 56.6% (n = 95) had confirmed *P.falciparum* infection with almost in a quarter (24.2%) another infection, mainly suspected lower respiratory tract infection (Figure 1). This later was also frequent among the 73 (43.4%) patients with a negative blood smear. Around ten percent (n = 44) of patients left the ward without an etiological diagnosis.

Anaemia was found in 355 (84.9%) children: 44 (10.5%) with severe anaemia and 114 (27.3%) with moderate anaemia. Malnutrition and respiratory distress were not associated with severe anaemia (*p* = 0.3). However, all patients with sickle cell disease had Hb level below 11 g/dL and were at higher risk of severe anaemia (29% *versus* 8.7% with normal hemoglobin; OR: 4.4[2.01-9.7] (*p* < 0.01)).

### Malaria patients

Approximately 23% (n = 95/418) of the participants had confirmed *P.falciparum* infection. They were significantly older (48 months) than those without malaria (18 months) (Table 2). The risk of having malaria was the highest between 37 and 120 months (aOR: 13.7[3.3-37.8] *p* < 0.01). Bednet use was associated with a reduced risk of malaria (17.0% *versus* 34.1%) (Table 3).

**Table 2:** Relationship between studied variables* and *P.falciparum *infection

	**Malaria slide-positive patients (N = 95)**	**Malaria slide-negative patients (N = 73)**	***p*-value**
**Age, months**	48 [24–72]	18 [9–36]	<0.01
**Duration of illness, days**	3 [2–4]	4 [2–6]	<0.01
**Temperature, °C**	39.0 [38.5-39.8]	38.8 [38–39]	<0.01
**Laboratory parameters**			
- Hb, g/dL	7.7 [5.8-9.7]	9.5 [7.3-10.5]	<0.01
- Leucocytes, 10^3^/μL	9.9 [7.1-12]	12.0 [8.7-18.0]	<0.01
- Platelets count, 10^3^/μL	89.0 [51.0-122.0]	298.0 [165.0-417.0]	<0.01

*: medians with interquartile ranges are presented.

**Table 3:** Logistic regression analysis of factors associated with risk of complicated malaria

**Variables**	**Malaria**	**OR**^**a **^**[95%CI]**^**b**^	***p***	**aOR**^**c **^**[95%CI]**	***p***
	**Prevalence**				
Age					
- ≥ 5 years (n = 86)	32 (37.2%)	2.6[1.5-4.3]	<0.01	2.5[1.3-4.7]	<0.01
- < 5 years (n = 332)	63 (19.0%)	1			
Bed net					
- users (n = 276)	47 (17.0%)	0.4[0.2-0.6]	<0.01	0.4[0.2-0.7]	<0.01
- non users (n = 129	44 (34.1%)	1			
Drug intake					
-Yes^**d**^ (n = 95)	41 (43.2%)	0.5[0.3-0.8]	<0.01	1.3[0.8-2.3]	0.3
-No^**d**^ (n = 323)	93 (28.8%)	1			
Pallor					
-Yes (n = 95)	66 (69.5%)	2.8[1.7-4.0]	<0.01	2.9[1.7-5.2]	<0.01
-No (n = 321)	143 (44.5%)	1			
Prostration					
-Yes (n = 95)	18 (18.9%)	2.3[1.2-4.3]	<0.01	3.6[0.0-40.8]	0.3
-No (n = 323)	30 (9.3%)	1			
Neurological symptoms^**f**^					
-Yes (n = 95)	39 (41.0%)	3.2[1.9-5.2]	<0.01	3.4[1.9-6.3]	<0.01
-No (n = 323)	60 (18.6%)	1			
Anaemia					
-Yes (n = 95)	86 (90.5%)	3.2[1.3-7.7]	<0.01	3.3[1.6-7.9]	<0.01
-No (n = 323)	269 (83.3%)	1			
Severe anaemia					
-Yes (n = 95)	14 (14.7%)	1.7[0.8-3.3]	<0.13	1.8[0.8-4.2]	0.1
-No (n = 323)	30 (9.3%)	1			
BCS ≤ 2					
-Yes (n = 95)	2 (2.1%)	---	---		
-No (n = 323)	0 (0.0%°	--	---		
Thrombopenia					
-Yes (n = 95)	80 (84.2%)	19.6[10.6-36.2]	<0.01	2.6[1.3-5.3]	<0.01
-No (n = 323)	69 (21.4%)	1			
VOC^**g**^					
-Yes (n = 95)	2 (2.1%)	0.2[0.02-0.7]	<0.01	0.1[0.0-0.5]	<0.01
-No (n = 323)	35 (10.8%)	1			

^a^: crude odds ratio; ^b^: 95% confidence interval; ^c^: adjusted odds ratio ^d^: malaria infected patients; ^e^: non infected patients;

^f^: see detailed symptoms in Table 1, ^g^ : vaso-occlusive crisis in patients with sickle cell disease.

At admission, infected patients presented more frequently with pallor (n = 66, 69.5%), convulsions (n = 25, 26.3%), vomiting (n = 22, 23.2%) and prostration (n = 18, 18.9%). Overall, 20 (21.1%) patients had severe malaria (SM) according to the WHO classification: two had CM, four presented a syndrom of respiratory distress and 14 (14.7% of malaria patients) with SMA. More than three quarters of SM patients (n = 11) were aged between 6 to 36 months old and the two children with CM were 48 and 60 months old respectively. Pallor and presence of neurological symptoms were associated with a higher risk of being infected (Table 3). Seizures were noticed more frequently in parasitaemic children (27.3% *versus* 10.2% in uninfected ones; OR: 3.3[1.8-5.9], *p* < 0.01).

The parasite density ranged from 700 to 622,000 p/μL with a median of 22,500[5,000-70,700]p/μL. Hyperparasitaemia was found in five children aged between 16 and 48 months. Most of the parasitemic patients were anaemic (90.5%) and the median Hb level was the lowest in the malaria group compared to the uninfected one. The same trend was observed with platelet count (Table 2). Anemia and thrombocytopenia were associated with a higher risk of having a malaria-positive blood slide (*p <* 0.01) (Table 3). The frequency of moderate and severe anemia was highest in infected patients (respectively 52.6% and 14.7%) compared to non malaria patients (respectively 34.5% and 10.5%) (*p <* 0.01). None of the HIV positive patients had malarial infection and only two children with malnutrition were infected.

### Treatment

#### Drug prescription

Approximately two thirds of patients (n = 261) were treated with antibiotics, only 115 having leucocytosis. Quinine was administered to 42.1% (n = 176) of patients, 81 (46.0%) of them were malaria blood slide negative. One patient without clinical and or biological malaria diagnosis was also treated with an antimalarial drug (Table 4). Among the 73 patients who benefited from antibiotic plus antimalarial treatment, 21 (28.8%) had a confirmed co-morbidity.

**Table 4:** Drug prescription for patients (n = 418) with positive or negative slides at CHL

**Drugs**	**Other diagnosis (n = 250)**	**Clinical diagnosis of malaria (n = 168)**		**Total (N = 418)**
		**Slide (+)ve (n = 95)**	**Slide (−)ve (n = 73)**	
**Antimalarial, n (%)**	1 (0.4)	59 (62.1)	43 (58.9)	103 (24.6)
**Antibiotic, n (%)**	188 (75.2)	0 (0.0)	0 (0.0)	188 (44.9)
**Antibiotic + Antimalarial**	9 (3.6)	36 (37.9)	28 (38.4)	73 (17.5)
**Other, n (%)**	52 (20.8)	0 (0.0)	2 (2.7)	52 (20.8)

#### Blood transfusion

A blood transfusion was given to 96 (27.0%) anaemic children: 41 had severe anemia and 55 had moderate anemia, their Hb level varied from 5.0 g/dL to 7.9 g/dL. Three patients with severe anemia were not transfused.

### Case fatality rate

Of the 418 patients, nine died, giving an overall case fatality rate of 2.1%. They were hospitalized for respiratory tract infection (n = 5), gastro-enteritis (n = 2), meningitis (n = 1) and malaria (n = 1). The case fatality rate for malaria was therefore of 1.0%; this patient presented respiratory distress and CM.

Death was associated with a higher median duration of illness before consultation, 7 [4.8-7.3] days *versus* 3 [2.0-5.0] days in the group of survivors (*p <* 0.01); and with a lower median age: 12 [3.0-14.5] months *versus* 24[12.0-48.0] months (*p <* 0.01). Six children were aged between 12 and 36 months and three were less than six months old. Case fatality rate among the less than five years old children was 2.7%.

## Discussion

Presumptive treatment of fever with antimalarial drugs is still frequent in public health settings of sub-Saharan Africa, even in countries where ACTs, bed nets, malaria diagnosis and IPT are freely provided to vulnerable population [8]. During the last years, the prevalence of fever due to malaria has been drastically reduced in several countries including Gabon [3,6]. There is an urgent need to better manage febrile illness, especially in children, to avoid high mortality due to misdiagnosis of other etiologies, and overtreatment of non confirmed malaria cases which leads to impairment of patient outcomes, waste of money and time, and increased risk of drug resistance [4,11]. Unfortunately, clinical diagnosis through the syndromic approach, according to the integrated management of children febrile illnesses, remains the main method for the diagnosis [11-13].

In Libreville, malaria declined from being the primary cause of hospitalization among febrile children admitted in the same pediatric ward in 2002, to representing the second cause six years later [14].

Hospital case management of seriously ill children is a challenge in middle and low income countries, mostly due to few resources for diagnosis and treatment, staff availability, and laboratory services [13,15]. Respiratory tract infections, gastro-enteritis, urinary infections and meningitis are the main evocated etiologies of severe febrile illness in hospitalized children either in west, east, central Africa and Asia [11,16-18]. Symptoms of these conditions always overlap with malaria, leading to difficult patient management, misuse of antibiotics and antimalarial and higher mortality rates [19,20]. Less than one quarter of children had co-morbidities, mostly malaria and lower respiratory tract infection, as reported in Kenya and Uganda [12,21]. Bacteremia was not confirmed when suspected in children included, although bacteria are the most frequently associated pathogens to *P.falciparum* infection, either through infection or co-incidental parasitaemia [20-22]. Beside bacteria, HIV infection and malnutrition are also frequent and responsible of life threatening conditions in malaria infected patients [22,23], this is not the case in the present study. Likewise, between 2005 and 2007, an increase rate of non-malarial and non-bacteremia fevers has been observed, with appearance of Chikungunya and Dengue infections, among other viral infections in Libreville [24]. Approximately half of the patients treated with antimalarial were blood smear negative, suggesting another undiagnosed cause of fever and the necessity of an improvement of clinical and laboratory diagnosis as well as training and supervision of health workers for a proper case management.

Globally, prostration, neurological symptoms like seizures and pallor are the main clinical forms of malaria among children with acute febrile illness as observed in other endemic countries [25,26]. Thrombocytopenia and low Hb level, both predictors of malaria, have also been found to be associated to a greater risk of being infected [27,28]. The increase of the frequency of *Plasmodium* infections with age observed since 2006 in outpatients was confirmed among the inpatients: children older than five years are 2.6 times more at risk for having complicated malaria. This suggests a change in the transmission profile of the disease as the prevalence of infection increases with age with decreasing transmission [26,29,30]. The median age of children with complicated malaria was 48 months compared to the non-infected (18 months). Moreover, cerebral malaria (CM), which has been shown to increase with age when the intensity of the transmission decreases, was observed in children aged more than 48 months old [30]. This was not the case six years earlier when 65% of CM was confined to children aged between 12 and 48 months. A recent meta-analysis showed that in areas with no seasonality, the median age of cases with CM increases with lowering transmission intensity (from 29 months in medium intensity areas to 49 months in low intensity areas), whereas the median age of cases of severe malarial anemia (SMA) did not significantly vary and remains concentrated among younger children [30]. In 2008, 75% of children with SMA were between 6 to 36 months old, and, in 2002, they were below 24 months [14]. The high burden of anemia (85%) and its multifactorial etiology are likely to be the reasons for the fact that SMA is the major clinical presentation of severe malaria (SM); it would therefore be difficult to use anaemia as marker for malaria transmission intensity characterization [11,30,31]. Indeed, despite the decline of complicated malaria prevalence observed in this study, the frequency of severe anemia has not drastically changed compared to 2001–2002 (9.4% in 2001–2002 and 10.5% in 2008), as well as that of SMA (14.3% in 2001–2002 and 14.7% in 2008) in Libreville [32]. This observation raises the problem of the complex aetiology of anaemia; moreover, it seems that *P.falciparum* infection often occurs in patients with chronic anaemia and increases its gravity [33].

Improvement of medical care is ongoing in Gabon, but most of the population cannot benefit from complementary examinations because of poor financial resources and lack of equipment at health centers. As an example, beside chest X-ray that is systematically prescribed in case of suspected respiratory tract infection, lactate levels cannot be measured at the CHL and material for blood and CSF cultures is always lacking. Therefore, it remains crucial to use clinical and biological signs to target malaria infected children for a rational use of antimalarial drugs in poor resource areas. Pallor, presence of neurological symptoms, BCS ≤ 2 and age >5 years, were predictors of malaria infection among children hospitalized for fever. These risk factors have also been found in children from other countries [16,34,35]. As always reported, Hb level below 8 g/dL and severe anemia are biological risk factors, thrombocytopenia also being a prognosis factor in children with parasitemia [28,35]. Other factors associated with poor outcome in SM children are low BCS, co-infection, HIV, bacteremia, hyperlactatemia, malnutrition and respiratory distress [10,20,34,36]. Children who died from SM had respiratory distress and CM, two clinical forms already reported as prognosis factors at Libreville in 2002.

Even if malaria diagnosis and hematological count were freely provided to patients, overdiagnosis (43%) and overtreatment with antimalarial drugs (46%) were common.

Although the low number of *P.falciparum*-infected patients, lethality was lower (1%) compared to that observed between 2001–2002 in the same paediatric ward (9%) and in 11 African countries (10.5%); probably due to i) the reduced cases of malaria, ii) a better case management because all malaria cases were microscopically confirmed and suspected co-morbidities treated with both antimalarial and antibiotics [37,38].

After the deployment of new preventive measures, decreased frequency of malaria hospital admissions and its various clinical presentations were observed [9]. However, despite an adequate coverage, the reduction of the malaria burden observed among children or pregnant women, during the first years of the deployment, was followed with either stabilization or, conversely, an increase in the frequency of *P.falciparum* infection [39-41]. The protective effect of these control strategies, confirmed here with the lower prevalence of malaria among bednet users, is well recognized. Regular monitoring should absolutely be implemented in Gabon where ITNs, ACTs and diagnostic tools coverage is still weak or lacking in the health centers from remote and peripheral areas.

This pilot study allowed the inclusion of an enough number of patients to provide reliable data on hospitalized febrile children. A higher number of inclusions would have probably not given more indications on febrile illness management in the paediatric ward, neither more clinical data. Indeed, the main limit of this study concerns the lack of additional investigations necessary to finalize the diagnosis. They are most of the time not performed because of poor financial resources of the parents. Furthermore, there are some limitations on using health facility–based data including the lack of a knowledge on variations in access to, or use of the facilities in the community, extend of real coverage by various interventions.

Nevertheless, hospital based studies provide accurate data on children febrile illness etiologies, though they cannot replace epidemiological surveys in the community [42]. The present data can be used for the characterization of the malaria epidemiology over time in a specific area. However, local data are needed for WHO guidance application and are lacking in several endemic countries, including Gabon.

## Conclusion

This study, and the one performed between 2001 and 2002, highlights the most likely causes of fever as well as a reduction in the frequency of *P.falciparum* infection in Gabon in 2008. These results can help for the design of an effective development of new guidelines for fever case management. Priority areas to achieve proper patient care are quality of diagnosis, treatment practices, availability of biological and radiographic diagnosis tools, introduction of malaria diagnosis RDTs to discriminate malarial to non malarial fevers and bring out rational use of drugs, health worker training and adherence to guidelines.

## Competing interests

The authors declare that they have no competing interests.

## Authors’ contribution

MKBA, EK, DPMM and MK were involved in design, implementation, planning of analysis. MKBA and EK were responsible for data analysis, writing up of the results. AEA, ARO and EEA were involved in patient care at hospital; and MKBA, DPMM and MK in the writing up and making critical revisions to the manuscript. MKBA is guarantor. All authors read and approved the final manuscript.

## Pre-publication history

The pre-publication history for this paper can be accessed here:

http://www.biomedcentral.com/1471-2334/12/216/prepub
